# Effect of Preoperative Administration of Intravenous Ferric Carboxymaltose in Patients with Iron Deficiency Anemia after Off-Pump Coronary Artery Bypass Grafting: A Randomized Controlled Trial

**DOI:** 10.3390/jcm12051737

**Published:** 2023-02-21

**Authors:** Hyo-Hyun Kim, Eun Hye Park, Seung Hyun Lee, Kyung-Jong Yoo, Young-Nam Youn

**Affiliations:** 1 Department of Cardiothoracic Surgery, Ilsan Hospital, National Health Insurance Service, Goyang-si 10444, Republic of Korea; 2 Division of Cardiovascular Surgery, Severance Cardiovascular Hospital, Yonsei University College of Medicine, Yonsei University Health System, Seoul 03722, Republic of Korea

**Keywords:** iron deficiency anemia, intravenous ferric carboxymaltose, off-pump coronary artery bypass grafting

## Abstract

Patients scheduled for cardiac surgery often have anemia and iron deficiency. We investigated the effect of the preoperative administration of intravenous ferric carboxymaltose (IVFC) in patients with iron deficiency anemia (IDA) who were due to undergo off-pump coronary artery bypass grafting (OPCAB). Patients who were due to undergo elective OPCAB between February 2019 and March 2022 who had IDA (*n* = 86) were included in this single center, randomized, parallel-group controlled study. The participants were randomly assigned (1:1) to receive either IVFC or placebo treatment. Postoperative hematologic parameters [hemoglobin (Hb), hematocrit, serum iron concentration, total iron-binding capacity, transferrin saturation, transferrin concentration, and ferritin concentration] and the changes in these parameters during the follow-up period were the primary and secondary outcomes, respectively. The tertiary endpoints were early clinical outcomes, such as the volume of mediastinal drainage and the need for blood transfusions. IVFC treatment significantly reduced the need for red blood cell (RBC) and platelet transfusions. Despite receiving fewer RBC transfusions, patients in the treatment group had higher levels of Hb, hematocrit, and serum iron and ferritin concentrations during weeks 1 and 12 after surgery. No serious adverse events occurred during the study period. Preoperative IVFC treatment in patients with IDA undergoing OPCAB improved the values of the hematologic parameters and iron bioavailability. Therefore, is a useful strategy for stabilizing patients prior to OPCAB.

## 1. Introduction 

Preoperative anemia affects approximately 30–60% of all patients undergoing major elective surgery and is associated with an increased risk of blood transfusion, poor recovery, and mortality [1,2]. The most common cause of anemia is iron deficiency, either due to nutritional deficiency or blood loss, which leads to a state of absolute iron deficiency, characterized by low iron stores [3,4]. International treatment guidelines recommend that patients undergoing surgery with an expected blood loss of ≥500 mL should be screened for anemia at least two weeks before the surgery, and those with anemia should be treated with intravenous iron [5,6]. 

Preoperative anemia increases the incidence of postoperative mortality and complications in patients undergoing open-heart surgery. In particular, it has been reported that preoperative anemia adversely affects the prognosis after coronary artery bypass surgery. It has also been reported that blood transfusion due to preoperative anemia increases the mortality rate after open-heart surgery. Off-pump coronary artery bypass grafting (OPCAB) that is performed without cardiopulmonary bypass shows better short-term postoperative performance than conventional coronary bypass surgery in patients with anemia. However, it has also been reported that postoperative transfusion can increase the rate of complications due to an increase in the levels of myocardial enzymes. 

These studies were conducted on non-anemic patients, included coronary bypass surgery and valve surgery, and involved data analysis without intergroup correctional analyses. Therefore, it was not possible to determine how the intravenous administration of iron before surgery affected the patient’s progress after surgery. Furthermore, as these studies targeted patients undergoing cardiopulmonary coronary bypass surgery, there is a paucity of data regarding cases of off-pump coronary bypass surgery. 

Thus, the prevalence rate of iron deficiency anemia in patients due to undergo OPCAB is unclear. Moreover, the use of intravenous iron to correct preoperative anemia and reduce the need for postoperative blood transfusions in patients before surgery is based on very low-quality evidence [7]. Therefore, we conducted a randomized controlled trial in order to evaluate the effects of preoperative intravenous ferric carboxymaltose (IVFC) on the postoperative hematologic parameters and transfusion requirements in patients who underwent coronary artery bypass surgery.

## 2. Material and Methods

### 2.1. Patient Selection and Definition of Groups

**Figure 1:**
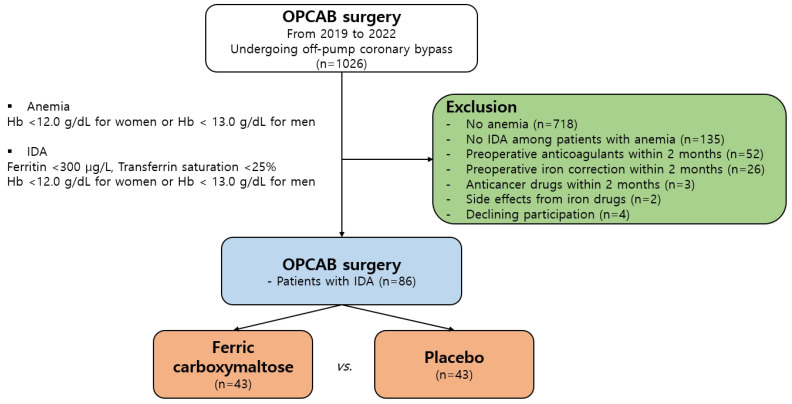
Flow diagram of study sample selection. OPCAB, off-pump coronary artery bypass grafting; IDA, iron deficiency anemia.

A total of 1026 consecutive patients who underwent OPCAB or minimally invasive direct coronary artery bypass (MIDCAB) between February 2019 and March 2022 were screened in this single-center, prospective, open-label, randomized controlled study. Patients aged ≥19 years with iron deficiency anemia (IDA) who underwent elective OPCAB or MIDCAB surgery, performed by one surgeon (Y. N. Y) who had carried out more than 1000 OPCAB surgeries, were included in this study. Patients without anemia (*n* = 718) and those without IDA (*n* = 135) were excluded from this study. Patients on preoperative anticoagulants (*n* = 52), iron correction treatment (oral or intravenous, *n* = 56), or drugs that could cause bone marrow suppression, such as anticancer drugs (*n* = 3), in the two months prior to surgery were excluded from the study. Patients with a history of hypersensitivity reactions, those who experienced side effects from iron drugs (*n* = 2), and those who declined participation (*n* = 4) were also excluded. Finally, we enrolled patients with IDA (ferritin < 300 μg/L, transferrin saturation < 25%, hemoglobin (Hb): <12.0 g/dL for women and <13.0 g/dL for men), which was determined preoperatively. The patients were randomly assigned to the IVFC group (intravenous (IV) Ferinject, *n* = 43) or the placebo group (IV normal saline, *n* = 43) (Figure 1).

### 2.2. Ethics Statements 

This study was approved by the Ethics Committee/Review Board of Severance Hospital, Republic of Korea (IRB number: 4-2019-0371) and was performed in accordance with the tenets of the 1964 Declaration of Helsinki and its later amendments. This study met the criteria of the WHO primary registry (ClinicalTrials.gov, NCT04898569). Preoperative informed consent was obtained from all the patients prior to the commencement of the study.

### 2.3. Surgical Technique

All the patients underwent OPCAB via median sternotomy. Heparin (0.7–1.0 mg/kg) was administered after full median sternotomy in order to achieve the target activated clotting time (ACT; >250 s). The left anterior descending artery was revascularized using the left internal mammary artery (LIMA) in situ. In several cases, the left-sided coronary system was revascularized using the right internal mammary artery (RIMA). Furthermore, the radial artery (RA) was combined with the LIMA or RIMA in order to achieve total arterial grafting. A saphenous vein graft (SVG) was the most frequently used secondary conduit to the circumflex and right coronary arterial territories in this study. For the patients in whom the aorta was used, the SVG or RA graft was anastomosed to the aorta using a HeartString device (MAQUET Holding B. V. & Co. KG, Rastatt, Germany). After completion of the anastomoses, the residual heparin was reversed with 1 mg of protamine for every 1 mg of heparin used for systemic heparinization. The pericardium was loosely closed after surgery, the midline incision was closed in layers, and two mediastinal drains were retained.

### 2.4. Iron Administration 

Participants were assigned to one of two groups in a 1:1 ratio using computer-generated permuted block randomization. The two groups were the IVFC group (*n* = 43) or the control group (*n* = 43). A surgeon who was not involved in data collection conducted the randomization and assigned the patients to the groups. The IVFC group received 1000 mg of IV iron (ferric carboxymaltose, Ferinject, Vifor Pharma, Baar, Switzerland), which was added to 0.9% sterile physiological saline solution in order to generate a total volume of 100 mL. The patients weighing less than 35 kg were administered an iron dose of no more than 500 mg. The solution was infused for 30 min 12 h before surgery. Both IVFC and the placebo were administered intravenously via a black infusion set, and the procedure was performed behind a screen in order to assure blinding of the patient. This was administered by a person who was not involved in data collection or data entering. 

### 2.5. Endpoints 

The primary endpoints were total mediastinal blood loss and transfusion counts during the admission period. The secondary endpoint was the volume of mediastinal drainage (blood loss on the operative day), the need for blood transfusion or surgical revision, the length of the intensive care unit (ICU)/hospital stay, and early clinical outcomes, including postoperative morbidity and mortality. The tertiary endpoint was the change in hematologic parameters during the follow-up period. All the laboratory values were obtained before surgery and 1 day, 1 week, and 3 months after surgery. Follow-up was completed for the whole cohort.

### 2.6. Sample Size Calculation 

Local audit data revealed that, in patients with a preoperative hemoglobin concentration of 10–13 g/dL, a difference in the Hb level of >1.0 g/dL between the two groups was considered clinically meaningful. In order to detect this difference with 80% power, we calculated that 40 participants were required, based on a two-tailed z-test, a significance level of 0.05, and 1:1 allocation. 

The hypothesis of this study was that ‘in patients with IVFC, the Hb level after 1 and 12 weeks of surgery would be no different from that of the control group without IVFC’. The level of significance (*α*) was 0.05, type 2 error (*β*) was 0.2, and the power of the test (1 − *β*) was 0.8. The elimination rate was 10%. 

The formula for calculating the sample size:

$$S_{p}^{2}=\frac{\left(n-1\right)S_{\chi }^{2}+\left(m-1\right)S_{\tau }^{2}}{n+m-2}$$

Common variance estimates:

n=2(Zα2+Zβ)2σ2(μc−μt)2 , Sp2=13.06.

Based on a previous study [8] and the above assumption, the sample size was calculated as 38 patients for each group, and the total target group consisted of 76 patients. A total of 86 participants were required considering the 10% drop-out rate. 

### 2.7. Statistical Analysis 

Randomization lists with a block size of 4 were generated at the start of the study using RandList software (DatInf GmbH, Tübingen, Germany). The categorical variables were summarized as frequencies and percentages and were compared using Fisher’s exact test or Pearson’s Chi-square analysis. The continuous variables were summarized as mean ± standard deviation, and were compared using the independent *t*-test or the Mann–Whitney *U* test. 

The statistical analysis was performed using a linear mixed-effects model (LMM) in order to analyze the overall changes in the hematologic parameters. The significance level was set at *p* < 0.05. The IBM Statistical Package for Social Sciences (version 25.0, IBM Corp., Armonk, NY, USA) and SAS System software (version 9.3, SAS Institute, Cary, NC, USA) were used for all the statistical analyses.

## 3. Results

### 3.1. Clinical Characteristics and Prevalence of Anemia 

Among all the screened patients (*n* = 1026), 30.0% (*n* = 308) had anemia before OPCAB surgery (32.8% men and 27.3% women). The overall mean Hb level was 12.7 ± 1.7 g/dL, and IDA was diagnosed in 173 patients (16.9%). The baseline demographic, clinical, and laboratory parameters of the randomized groups are summarized in Table 1. The mean age of the patients at the time of surgery was 71.4 ± 8.8 years, and they were predominantly males (61/86, 70.9%). Before surgery, there were no significant differences in the Hct and Hb levels between the two groups (*p* = 0.229 and *p* = 0.586, respectively).

### 3.2. Operative Data 

The patients underwent either elective OPCAB (*n* = 79, 91.9%) or MIDCAB (*n* = 7, 8.1%). The baseline ACT and intraoperative requirements of heparin and protamine did not differ significantly between the two groups. Intraoperative estimated blood loss did not differ significantly between the groups, and one (2.3%) individual in each group underwent reoperation for mediastinal bleeding (Table 1).

### 3.3. Changes in Hematologic Parameters 

Table 2 summarizes the time–effect comparison of the hematologic parameters immediately after surgery and during the follow-up period. One week after surgery, the levels of Hb, hematocrit, serum iron concentration, transferrin saturation, transferrin concentration, and ferritin concentration were higher in the IVFC group than in the placebo group. Twelve weeks after surgery, the levels of Hb, Hct, serum iron concentration, and ferritin concentration remained higher in the IVFC group than in the placebo group.

### 3.4. Linear Mixed-Effects Model for Changes in Hematologic Parameters 

**Figure 2:**
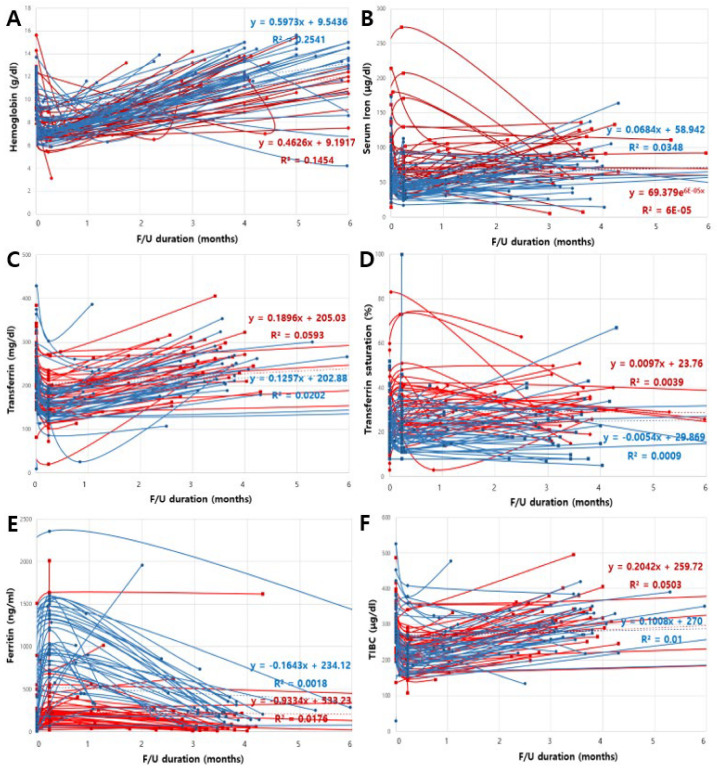
Spaghetti plots of the changes in the hematologic parameters over time after coronary artery bypass in the intravenous ferric carboxymaltose (IVFC) group (—) vs. the placebo group (—) for patients receiving preoperative intravenous iron. (**A**) Hemoglobin, (**B**) Serum iron concentration, (**C**) transferrin concentration, (**D**) transferrin saturation, (**E**) ferritin concentration, and (**F**) total iron-binding capacity (TIBC). F/U, follow-up.

Individual trees of the distinct hematologic parameters were measured in order to evaluate the early changes after surgery (Figure 2). The LMM also indicated that the parameters changed over time. In the IVFC group, the transferrin concentration exhibited the steepest increase (0.1257/month; *p* = 0.020), followed by the TIBC (0.1008/month; *p* = 0.010) and serum iron concentration (0.0684/month; *p* = 0.0348). The LMM revealed statistically significant between-group differences in changes in the serum iron concentration, TIBC, transferrin saturation, transferrin concentration, and ferritin concentration during the follow-up period.

### 3.5. Transfusions and Early Clinical Outcomes 

There were no differences in intraoperative blood loss (*p* = 0.239, Table 1) or blood loss on postoperative days 1 and 2 (*p* = 0.851 and *p* = 0.358, respectively; Table 3). However, the need for RBC and platelet transfusions was lower in the IVFC group than in the placebo group.

The median follow-up time was 5.6 months (interquartile range, 3.9–9.2 months). During the follow-up period, the overall mortality rates in the IVFC and placebo groups were 2.3% and 4.7%, respectively. The length of postoperative stays in the ICU and the hospital did not differ between the two groups (*p* = 0.093 and *p* = 0.520, respectively). No adverse reactions related to ferric carboxymaltose were observed during the study period.

## 4. Discussion

In this randomized clinical trial, preoperative IDA was commonly found in the patients who were due to undergo elective OPCAB. We used an LMM to analyze the changes in hematologic parameters according to the administration of IVFC before OPCAB surgery. The preoperative administration of IV iron maintained Hb levels in the postoperative period as it increased hematopoiesis and iron bioavailability. Furthermore, we discovered that IVFC reduced the need for blood transfusions after cardiac surgery. These findings suggest that physicians can use IVFC to manage preoperative iron deficiency. 

### 4.1. Prevalence of Preoperative Anemia in Patients Undergoing OPCAB

The prevalence of preoperative anemia was reported to be 30% in patients who were scheduled to undergo various types of surgery in the European Outcomes Studies (EUROS) [9]. In 2011, Hung et al. reported that the prevalence of anemia in patients who were scheduled to undergo cardiac surgery between 2008 and 2009 at single center in the United Kingdom was 64.4% [10]. Klein et al. reported that the incidence of anemia in patients undergoing cardiac surgery was 31% and that anemia was related to mortality [11]. Our investigation showed that 30.0% of the patients scheduled to undergo OPCAB surgery at our center had preoperative anemia, and that 16.9% had IDA.

### 4.2. Effect of IV Iron Treatment on Hematologic Parameters 

Low levels of serum ferritin before IVFC and transferrin saturation are useful proxies for the amount of iron stored within the body. A study indicated that laboratory results typically revealed reduced levels of serum ferritin and iron and increased levels of serum transferrin and TIBC values during progressive iron depletion [12]. In our study, similar results were observed in the control group at 1 week and 12 weeks postoperatively, but not in the IVFC group. This may be because IVFC improves iron biochemical outcomes and the hematopoietic response to severe anemia, as well as providing long-term normalization of Hb levels.

### 4.3. Benefit of IV Iron on Hb Levels and Need for RBC Transfusion 

The conductors of the IRONMAN trial reported a difference of 0.7 g/dL in the level of Hb 15–18 days after IV iron treatment [13]. Khalafallah et al. reported a mean difference of 0.78 g/dL in the level of Hb between patients receiving IV iron treatment and those not receiving IV iron treatment after 4 weeks [8]. In the present study, the mean difference in the level of Hb between the groups was 1.3 g/dL after 1 week and 1.6 g/dL after 12 weeks. Therefore, we demonstrated that the effects of IV iron treatment lasted 12 weeks. As predicted, the rates and amounts of RBC transfusion differed between the two groups. 

This study suggests the use of IVFC treatment for IDA in patients due to undergo elective OPCAB or MIDCAB. This treatment plays a critical role in establishing hematologic stability in the early postoperative period and in maintaining this stability for up to 12 weeks after surgery. Therefore, the preoperative optimization of serum iron would result in a reduced need for transfusions and could be an acceptable treatment option. 

### 4.4. Study Limitations 

The main limitation of this study was that we did not perform a cost–benefit analysis. It could be argued that the costs of the intervention may not be offset by the savings achieved from a reduction in transfusion rates. Such an analysis should be conducted in future studies. Furthermore, apart from IDA, anemias can develop from other causes, including chronic diseases. Lastly, we did not evaluate the effects of improved hemoglobin concentration on the quality of life or functional capacity of the patients after surgery. 

## 5. Conclusions 

**Figure 3:**
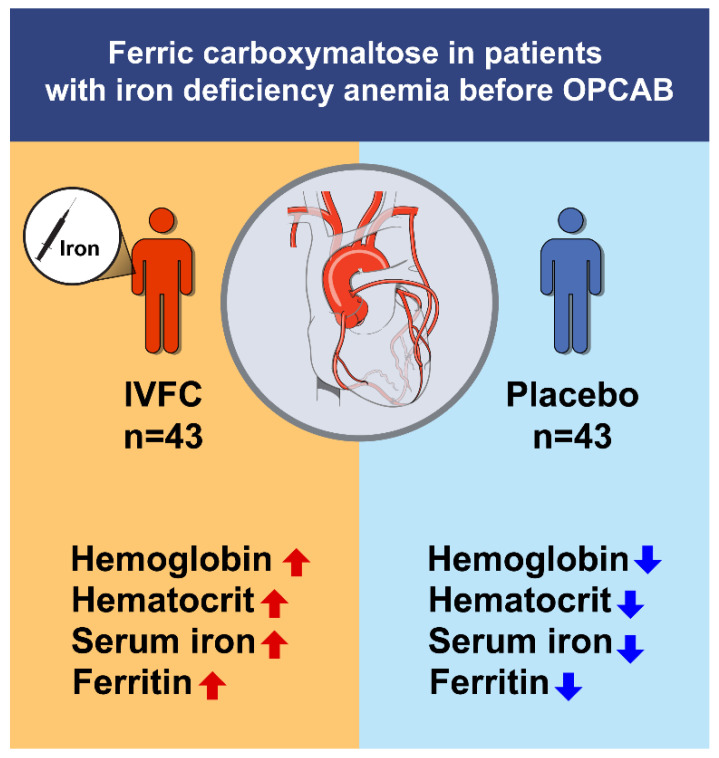
Infographic: Preoperative iron administration improved the postoperative recovery of hematologic parameters.

We have demonstrated that IVFC can increase postoperative Hb concentrations and reduce the need for blood transfusions in patients with IDA who are scheduled to undergo OPCAB surgery. Furthermore, the benefits of the administration of a single dose of IVFC may persist for up to 12 weeks after surgery (Figure 3).

## Figures and Tables

**Table 1:** Baseline characteristics and operative data for patients with preoperative iron deficiency anemia.

Variables	IVFC (*n* = 43)	Placebo (*n* = 43)	*p*-Value
Sex			0.812
Male, *n* (%)	31 (72.1)	30 (69.8)	
Female, *n* (%)	12 (27.9)	13 (30.2)	
Age, mean ± SD, years	70.6 ± 8.3	72.2 ± 9.2	0.383
BMI, mean ± SD, kg/m^2^	23.5 ± 2.6	22.8 ± 5.8	0.503
Preoperative Hb, mean ± SD, g/dL	11.3 ± 1.3	11.1 ± 1.7	0.586
Preoperative Hct, mean ± SD, %	42.0 ± 48.5	33.0 ± 5.2	0.229
Hypertension, *n* (%)	32 (74.4)	37 (86.0)	0.279
Diabetes mellitus, *n* (%)	31 (72.1)	20 (46.5)	0.028
CKD (grades III–V), *n* (%)	4 (9.3)	4 (9.3)	0.999
CVA history, *n* (%)	5 (11.6)	4 (9.3)	0.999
Atrial fibrillation, *n* (%)	2 (4.7)	2 (4.7)	0.999
Preoperative LVEF, mean ± SD, %	54.6 ± 9.6	56.8 ± 11.2	0.493
Previous MI, *n* (%)	3 (7.0)	2 (4.7)	0.828
Previous PCI, *n* (%)	4 (9.3)	2 (4.7)	0.714
History of peripheral arterial disease, *n* (%)	4 (9.3)	7 (16.3)	0.520
Type of operation, *n* (%)			0.433
OPCAB	38 (88.4)	41 (95.3)	
MIDCAB	5 (11.6)	2 (4.7)	
Preoperative ACT, mean ± SD, s	133.1 ± 15.5	141.1 ± 15.4	0.160
Peak ACT, mean ± SD, s	232.8 ± 33.5	255.8 ± 37.3	0.237
Heparin, mean ± SD, U	6046.3 ± 4994.2	5482.9 ± 1236.7	0.485
Protamine, mean ± SD, mg	22.5 ± 3.7	21.5 ± 4.2	0.462
Total operative time, mean ± SD, min	249.8 ± 33.3	252.1 ± 43.0	0.583
EBL during surgery, *n* (%)			0.239
<100 mL	13 (30.2)	12 (27.9)	
100–500 mL	15 (34.9)	9 (20.9)	
>500 mL	15 (34.9)	22 (51.2)	
Re-operation for mediastinal bleeding, *n* (%)	1 (2.3)	1 (2.3)	0.999

IDA, iron deficiency anemia; IVFC, intravenous ferric carboxymaltose; SD, standard deviation; BMI, body mass index; Hct, hematocrit; Hb, hemoglobin; CKD, chronic kidney disease; CVA, cerebrovascular accident; LVEF, left ventricular ejection fraction; MI, myocardial infarction; PCI, percutaneous coronary intervention; OPCAB, off-pump coronary artery bypass grafting; MIDCAB, minimally invasive direct coronary artery bypass; ACT, activated clotting time; EBL, estimated blood loss.

**Table 2:** Comparison of hematologic parameters.

Variables	IVFC (*n* = 43)	Placebo (*n* = 43)	*p*-Value	IVFC (*n* = 43)	Placebo (*n* = 43)	*p*-Value	IVFC (*n* = 43)	Placebo (*n* = 43)	*p*-Value
	1 Day			1 Week			12 Weeks		
Hemoglobin (g/dL)	11.3 ± 1.3	11.1 ± 1.7	0.993	8.3 ± 6.2	7.0 ± 0.8	0.025	13.0 ± 1.6	11.4 ± 2.2	<0.001
Hematocrit (%)	42.0 ± 48.5	33.0 ± 5.2	0.278	23.5 ± 11.1	20.8 ± 2.5	0.085	41.2 ± 4.4	30.6 ± 9.9	0.032
Platelet counts (10^3^/μL)	272.0 ± 33.6	217.4 ± 61.3	0.987	166.4 ± 74.1	167.0 ± 50.0	0.920	218.5 ± 63.5	203.1 ± 70.3	0.195
Serum iron (μg/dL)	71.8 ± 43.3	70.7 ± 33.2	0.861	81.4 ± 51.7	43.8 ± 16.8	0.035	81.6 ± 30.9	69.0 ± 30.6	0.048
TIBC (μg/dL)	300.6 ± 75.5	287.8 ± 71.2	0.667	229.7 ± 65.3	276.2 ± 36.3	0.125	289.8 ± 65.5	268.6 ± 50.8	0.215
Transferrin saturation (%)	25.7 ± 13.2	25.2 ± 11.1	0.991	33.4 ± 15.9	25.7 ± 35.8	0.049	30.2 ± 10.4	26.3 ± 17.1	0.296
Transferrin (mg/dL)	231.9 ± 68.0	228.0 ± 61.5	0.447	571.1 ± 260.5	273.6 ± 49.8	<0.001	230.1 ± 55.4	236.4 ± 65.3	0.824
Ferritin (ng/mL)	97.9 ± 79.0	172.4 ± 273.0	0.092	1173.2 ± 858.4	169.9 ± 266.5	<0.001	381.0 ± 393.1	133.0 ± 413.2	0.035

Data are expressed as mean ± SD from a linear mixed-effects model. POD, postoperative day. The adjusted *p* value is the Bonferroni-corrected *p*-value. IVFC, intravenous ferric carboxymaltose; TIBC, total iron-binding capacity.

**Table 3:** Early clinical outcomes.

Variables	IVFC (*n* = 43)	Placebo (*n* = 43)	*p*-Value
Mediastinal drainage 24 h postoperatively, mean ± SD, mL	413.2 ± 236.5	421.9 ± 186.5	0.851
Mediastinal drainage 24–48 h postoperatively, mean ± SD, mL	300.6 ± 115.0	327.4 ± 151.5	0.358
Postoperative blood transfusion rates, *n* (%)	25 (58.1)	31 (72.1)	0.258
Packed RBC, *n* (%)	8 (18.6)	12 (27.9)	0.046
Packed RBC, mean ± SD, mL	115.9 ± 55.3	153.6 ± 88.2	0.037
FFP, *n* (%)	23 (53.5)	24 (55.8)	0.829
FFP, mean ± SD, U	1.2 ± 1.2	1.4 ± 1.5	0.474
Platelets, *n* (%)	4 (9.3)	10 (23.3)	0.049
Platelets, mean ± SD, U	0.6 ± 1.8	1.7 ± 3.3	0.043
ICU stay, mean ± SD, days	2.8 ± 1.4	2.3 ± 1.6	0.093
Hospital stay, mean ± SD, days	8.2 ± 13.8	7.5 ± 10.3	0.520
Adverse drug events ^†^, *n* (%)	0 (0)	0 (0)	0.999
In-hospital mortality, *n* (%)	0 (0)	2 (4.7)	0.135
Overall mortality, *n* (%)	1 (2.3)	2 (4.7)	0.241

IVFC, intravenous ferric carboxymaltose; SD, standard deviation; RBC, red blood cell; FFP, fresh frozen plasma; ICU, intensive care unit. ^†^ Unintended, harmful events, including unscheduled hospital admissions and events attributed to the use of medicines.

## Data Availability

All data generated or analyzed during this study are included in this published article and its Supplementary Information Files.

## Supplementary Materials

The following supporting information can be downloaded at: https://www.mdpi.com/article/10.3390/jcm12051737/s1, Table S1: CONSORT 2010 checklist of information to include when reporting a randomised trial*.
